# Fabrication and in vitro biological properties of piezoelectric bioceramics for bone regeneration

**DOI:** 10.1038/srep43360

**Published:** 2017-02-27

**Authors:** Yufei Tang, Cong Wu, Zixiang Wu, Long Hu, Wei Zhang, Kang Zhao

**Affiliations:** 1 Department of Materials Science and Engineering, Xi’an University of Technology, Xi’an 710048, PR China; 2 Institute of Orthopaedics, Xi’jing Hospital, The Fourth Military Medical University, Xi’an 710032, PR China

## Abstract

The piezoelectric effect of biological piezoelectric materials promotes bone growth. However, the material should be subjected to stress before it can produce an electric charge that promotes bone repair and reconstruction conducive to fracture healing. A novel method for *in vitro* experimentation of biological piezoelectric materials with physiological load is presented. A dynamic loading device that can simulate the force of human motion and provide periodic load to piezoelectric materials when co-cultured with cells was designed to obtain a realistic expression of piezoelectric effect on bone repair. Hydroxyapatite (HA)/barium titanate (BaTiO_3_) composite materials were fabricated by slip casting, and their piezoelectric properties were obtained by polarization. The *d*_33_ of HA/BaTiO_3_ piezoelectric ceramics after polarization was 1.3 pC/N to 6.8 pC/N with BaTiO_3_ content ranging from 80% to 100%. The *in vitro* biological properties of piezoelectric bioceramics with and without cycle loading were investigated. When HA/BaTiO_3_ piezoelectric bioceramics were affected by cycle loading, the piezoelectric effect of BaTiO_3_ promoted the growth of osteoblasts and interaction with HA, which was better than the effect of HA alone. The best biocompatibility and bone-inducing activity were demonstrated by the 10%HA/90%BaTiO_3_ piezoelectric ceramics.

Hydroxyapatite (HA) is widely used in the fabrication of bone repair materials because of its similarity to the inorganic components of human bone and its biological activity^1^,^2^. HA has been used in numerous studies to prepare porous scaffolds for bone substitute materials^3^,^4^. The nano-HA coating on the surface of metal (such as Ti alloy) was also prepared based on the bionic human bone composition^5^. The surface and volume effects of the nanomaterials are easy to produce and stabilize using other atoms to promote bone cell growth. Performance improvement of HA by inducing the collagen, growth factor, and other bio-inspired organic compounds has also been studied^6^,^7^. Existing studies have continuously optimized the composition and structure of HA bioactive materials, but the biological properties of these materials are based on the osteoacusis of HA^8^,^9^. Numerous clinical trials have shown that the bone induction of HA was low. The growth cycle of new bones induced with HA usually takes half a year to one year, which is not conducive to the early activities of patients after surgery especially those in the middle- and old-age groups^10^,^11^,^12^.

The human bone tissue is also a type of piezoelectric material, that is, the composition of the human body can produce biological electricity owing to the electron displacement of the local electric field produced by deformation force^13^,^14^,^15^. This electrical activity will affect many biochemical reactions in the body, and energy conversion can be achieved in the process during these biochemical reactions. The effect of external force on the human body is transmitted by biological electricity. Biological electricity also affects the growth factor and extracellular matrix, which in turn can affect bone reconstruction and repair^16^. On the basis of the piezoelectric properties of biological tissues, some works used piezoresponse force microscopy (PFM) to investigate localized piezoelectric behavior^17^,^18^. In addition, in the aspect of piezoelectric effect, the use of BaTiO_3_ as a hard tissue replacement material has been reported^19^, and *in vivo* animal experiments have shown its good biocompatibility. BaTiO_3_ after polarization can lead to calcium phosphate deposit^20^. The surface cell adhesion and proliferation of polarizing HA were also investigated. Findings indicate that the surface of polarized HA can bond inorganic ions and organic cell adhesive proteins, thereby resulting in accelerated mineralization and cell adhesion and proliferation on the polarized HAp surface^21^,^22^. BaTiO_3_, CaTiO_3,_ and HA composites can also be used as bone repair materials, and an *in vitro* cell experiment found that the introduction of piezoelectric phase could promote the adhesion and proliferation of mouse fibroblast L929 and human osteoblast SaOS2^23^. HA/BaTiO_3_ composites can also be made into porous structures^24^, and the piezoelectric effect increases with the increase of BaTiO_3_ content. Moreover, *in vitro* experiments showed that composites had no cytotoxicity and had good biological activity. All these studies and findings show that the introduction of piezoelectric materials can induce bone growth^25^,^26^,^27^. During *in situ* loading on the animal bone, the mineralization behavior in simulated body fluid was observed, thereby indicating that the piezoelectric effect of bone promotes mineralization^28^,^29^. However, biological experiments have not considered how the growth of bone cell is promoted by the effect of piezoelectric material with physiological loading (cycle loading). In particular, the piezoelectric properties of the samples are actually in the residual polarization rather than the piezoelectric effect of the piezoelectric material.

A novel method for *in vitro* experiment of biological piezoelectric material with physiological loading is presented in this paper. A dynamic loading device for piezoelectric bioceramics based on the human activity cycle was designed; this device can mimic human motion to a given physiological loading to materials periodically when co-cultured with cells and can express the influence of the piezoelectric effect for bone repair accurately. Composite materials with different piezoelectric properties were obtained by adjusting the ratio of HA and BaTiO_3_. During simulated body fluid (SBF) immersion and co-culture with cells, physiological loading was applied on the surface of HA/BaTiO_3_ composites quantitatively and periodically. The deposition of HA and the adhesion of osteoblasts on the surface of composites with and without cycle loading were observed, and the proliferation and activity of osteoblasts were characterized.

## Results and Discussion

### Morphologies and phase composition of HA/BaTiO_3_ composite materials

**Figure 1:**
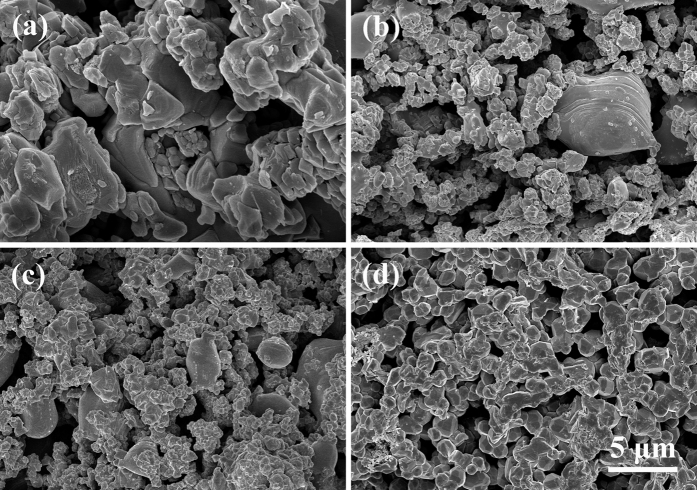
Morphologies of HA/BaTiO_3_ composite materials with various compositions. (**a**) 100% BaTiO_3_; (**b**) 90% BaTiO_3_/10% HA; (**c**) 80% BaTiO_3_/20% HA; (**d**) 100% HA.

Figure 1 shows the morphologies of HA/BaTiO_3_ composite materials with various compositions. After sintering, the grain growth of each composite group was uniform, and a gap was present between the grains. Morphologies of 100% BaTiO_3_ and 100% HA showed similar grain size without excessive growth, which had a positive effect on the performance of biological materials to ensure stability. The BaTiO_3_ particles accounted for the majority in 90% BaTiO_3_/10% HA and 80% BaTiO_3_/20% HA composites. BaTiO_3_ particles in the composite were connected to form the network, which was conducive for obtaining the overall piezoelectric effect^30^.

**Figure 2:**
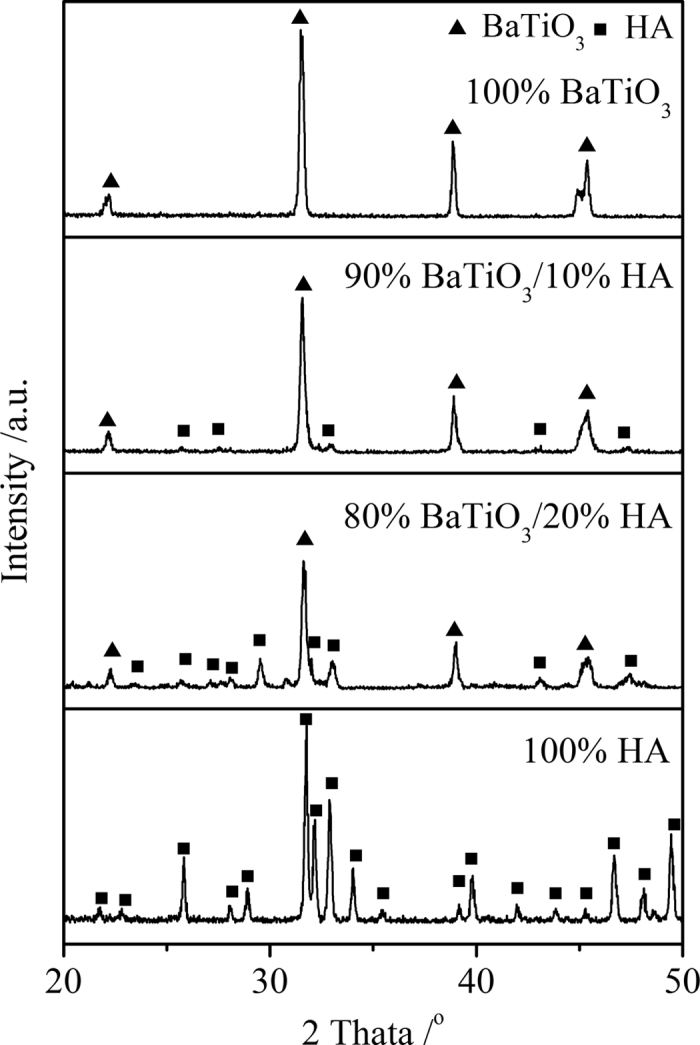
XRD patterns of HA/BaTiO_3_ composite materials with various compositions.

Figure 2 shows the X-ray diffraction (XRD) patterns of HA/BaTiO_3_ composite materials with various compositions sintered at 1250 °C. Diffraction peaks of 100% BaTiO_3_ after sintering were in agreement with the standard JCPDS card no. 5–626, and the diffraction peak of 100% HA were in agreement with JCPDS 9–432, thereby indicating that the phase composition of BaTiO_3_ and HA did not change during sintering. Compared with the 100% BaTiO_3_ group, some diffraction peaks of BaTiO_3_/HA composite materials appeared weak, thereby indicating the presence of HA. The diffraction peak of HA increased with HA content.

### Piezoelectric and mechanical properties of HA/BaTiO_3_ composite materials

**Figure 3:**
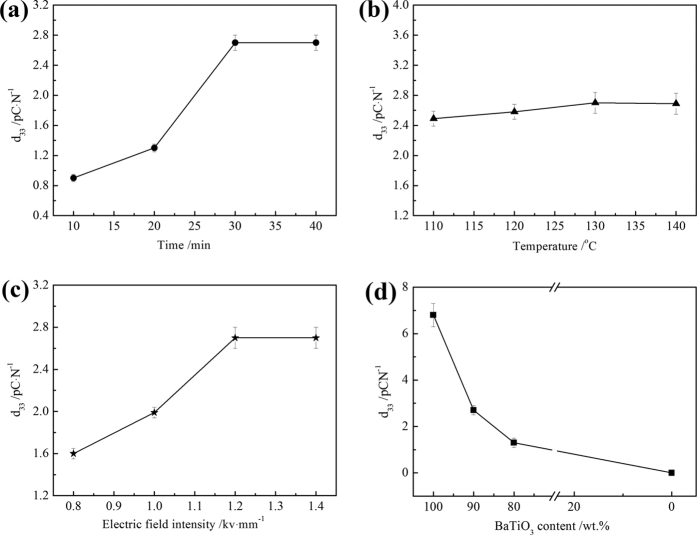
Effects of polarization process and composition on the d_33_ of BaTiO_3_/HA piezoelectric bioceramics. (**a**) Polarization time; (**b**) Polarization temperature; (**c**) Polarized electric field intensity; (**d**) BaTiO_3_ content. Plots in (**a**–**c**) are measured using 90% BaTiO_3_/10%HA composite samples.

Figure 3(a) shows the effects of polarization time on the piezoelectric coefficient (*d*_33_) of 90%BaTiO_3_/10%HA piezoelectric bioceramics. The *d*_33_ of BaTiO_3_/HA piezoelectric ceramics initially increased with the increasing polarization time and then remained constant. The polarization of piezoelectric ceramics is the process in which the spontaneous polarization direction of the electric domain turns to the direction of the external electric field. The steering and orientation of the electric domain were gradually completed^31^,^32^. With the increase of the polarization time, the electric domain gradually overcame resistance to orientation, and the *d*_33_ increased. However, when the polarization time is more than 30 min, the *d*_33_ remained constant because the interfacial polarization, ion displacement polarization, inversion of the 180° domain, and shift of the 90° domain were completed. Figure 3(b) shows the influence of polarization temperature on the piezoelectric constant of 90%BaTiO_3_/10%HA composites. The *d*_33_ significantly increased with the increasing polarization temperature but remained constant after the temperature reached 130 °C^33^. The formation of electric domains has a tendency to follow the preferential orientation of the external electric field. In the phase transition, the newly formed electric domain orientation along the direction of the external electric field is more likely to be easier than that of the electric domain that has been formed. Obtaining a high piezoelectric constant is easier at a high temperature than at room temperature (25 °C). However, 130 °C is the Curie point temperature of BaTiO_3_, and the *d*_33_ did not increase at higher polarization temperatures. Figure 3(c) shows the optimization of polarized electric field intensity of 90%BaTiO_3_/10%HA composites. The *d*_33_ increased with increasing polarized electric field intensity. The *d*_33_ maintained a constant maximum value of 2.7 pC·N^−1^ when the polarization field strength exceeded 1.2 kV·mm^−1^. The polarization and ferroelectric to paraelectric phase transition occurred at the same time. The electric domain is the preferred orientation in the formation process, and it arranged along the direction of the external electric field. However, the preferred orientation of the electric domains was disturbed and changed because the grains were active at high temperature^34^. The high electric field intensity could decrease the direction changes of the electric domain; hence, a lower limit of the electric field intensity exists. Through the optimization of different polarization conditions, the optimal polarization process was obtained for the polarization time of 30 min, polarized electric field intensity of 1.2 kV·mm^−1^, and polarization temperature of 130 °C. Other composite materials (BaTiO_3_ content is 80% or 100%) showed the same variation. To ensure comparability between the four groups, the optimized polarization process was used for the polarization of all samples; the results are shown in Fig. 3(d). With the reduction of the BaTiO_3_ content, the *d*_33_ decreased from 6.8 to 1.3 pC·N^−1^, and that of the 100% HA group was 0. The piezoelectric coefficient *d*_33_ of 90% BaTiO_3_/10%HA composites is close to that of the human bone range from 0.7–2.3 pC/N^35^.

Table 1 shows the compressive strength of HA/BaTiO_3_ with different BaTiO_3_ contents. Note that 100% BaTiO_3_ and 100% HA groups have high compressive strengths, namely, 28.4 and 21.8 MPa, respectively. The compressive strengths of HA/BaTiO_3_ composites are lower than those of pure BaTiO_3_ or HA because HA dispersed in the BaTiO_3_ phase and chemical reaction did not occur in the sintering process but in the formation of a physical combination. Moreover, HA hindered the sintering densification process of BaTiO_3_.

### *In vitro* characterization of HA/BaTiO_3_ piezoelectric bioceramics without loading

**Figure 4:**
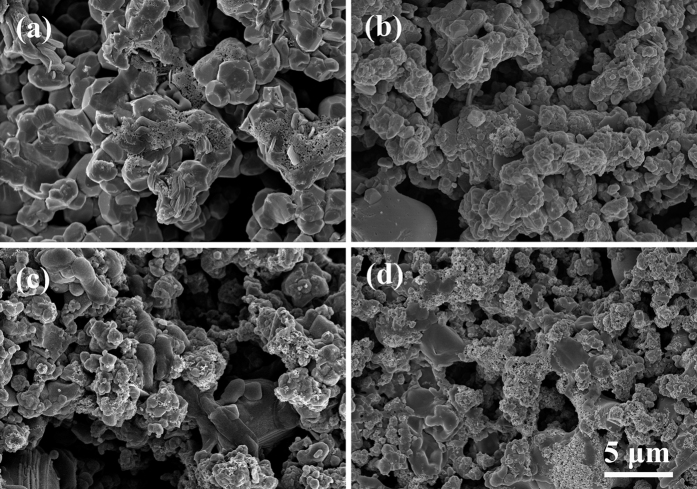
Morphologies of HA/BaTiO_3_ piezoelectric bioceramics after SBF immersion for 7 days without loading. (**a**) 100% BaTiO_3_, (**b**) 90% BaTiO_3_/10% HA, (**c**) 80% BaTiO_3_/20% HA, (**d**) 100% HA.

The formation of bone-like apatite by Ca and P ion deposition in the body fluid is an important advantage of the bioactive materials for use as bone implants^36^,^37^. Bone-like apatite plays an important role in the formation of new bone because it serves as the transition and bonding layer between the implant material and new bone. The absorption of ions and proteins in the blood is beneficial to the adhesion of bone cells and provides a suitable surface for the growth of new tissue^38^. Negative surface morphologies of HA/BaTiO_3_ piezoelectric bioceramics after 7 days of immersion in SBF are shown in Fig. 4. Some particles were deposited on the surface of the four groups after soaking. The particle size was much smaller than that of the grains shown in scanning electron microscopy (SEM) photos before soaking. The particles can be speculated to be bone-like apatite particles^39^. However, in the four groups of materials, the deposition amount of HA was more than that in the group that contained BaTiO_3_. This finding shows that the biocompatibility of HA is higher than that of BaTiO_3_, and no obvious difference exists between the three groups that contained BaTiO_3_. HA was degraded slowly after contact with SBF, few calcium and phosphate ions on the HA surface was formed. but calcium and phosphate ions in SBF will be enriched to the surface of HA. When the Ca ion concentration reached the critical value of the bone-like apatite nucleation, the nucleation formed and grew spontaneously^40^. However, the amount of deposition and its induction ability were not yet determined.

**Figure 5:**
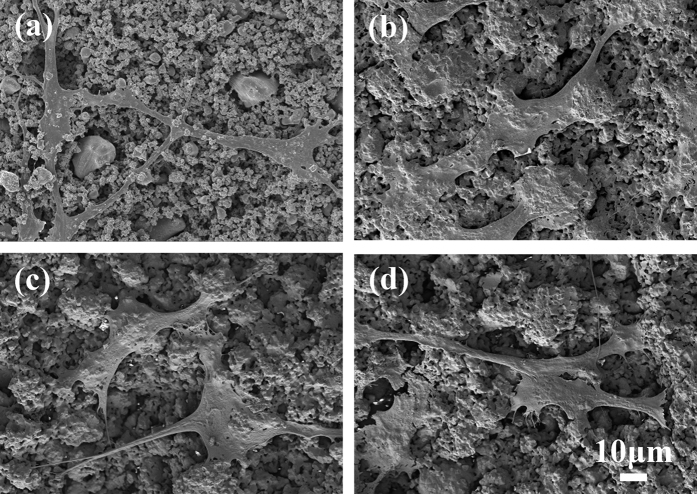
Morphologies of HA/BaTiO_3_ piezoelectric bioceramics without loading after co-culture with osteoblast cells for 3 days. (**a**) 100% BaTiO_3_, (**b**) 90% BaTiO_3_/10% HA, (**c**) 80% BaTiO_3_/20% HA, (**d**) 100% HA.

The morphology of osteoblasts on the surface can directly reflect the biological activity of the material^41^. Figure 5 shows the growth of osteoblasts on the negative surface of HA/BaTiO_3_ piezoelectric bioceramics without loading after co-culture for 3 days. Osteoblasts are polygonal and distributed on the material surface, and the pseudopodium is connected with piezoelectric bioceramics and neighboring osteoblasts. The cells associated with each sample showed excellent growth, including good cell morphology, extracellular secretions, and intercellular connections. An insignificant difference in the growth of osteoblasts on the surface of HA/BaTiO_3_ piezoelectric bioceramics with different contents was observed, thereby showing that the BaTiO_3_ also had biological activity. HA is a widely used bone substitute material, and its biological activity is generally recognized. A comparison of the HA group with the other three groups that contained BaTiO_3_ demonstrated no obvious differences in osteoblast growth.

**Figure 6:**
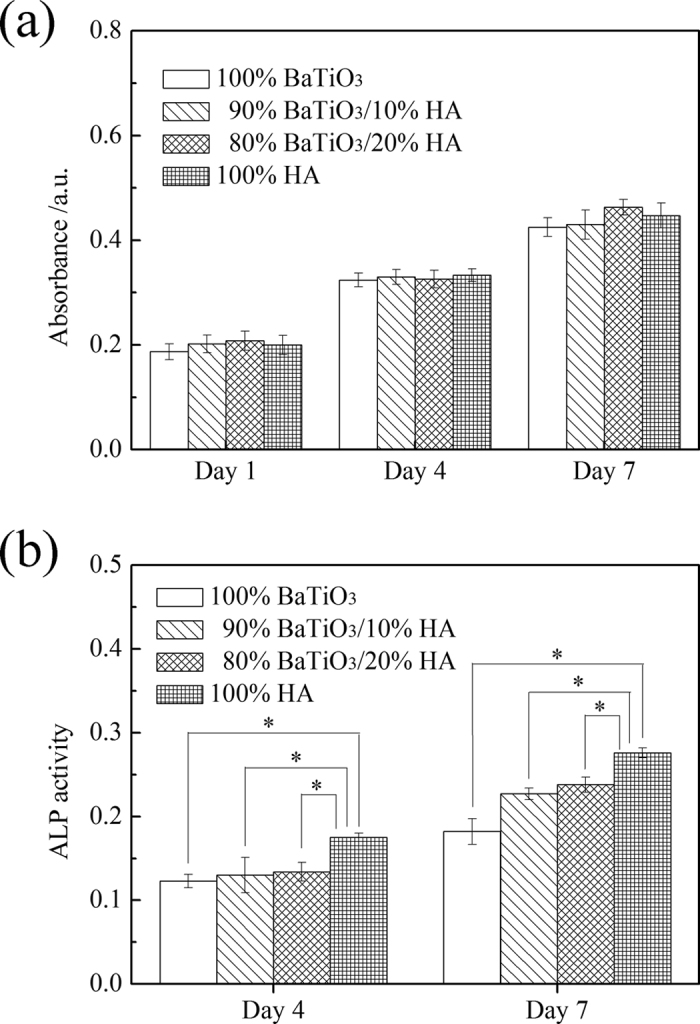
MTT assay and ALP activity of HA/BaTiO_3_ piezoelectric bioceramics without loading after co-culture with osteoblast cells (p < 0.05). (**a**) MTT assay; (**b**) ALP activity.

Figure 6(a) shows the MTT assay of HA/BaTiO_3_ piezoelectric bioceramics without loading after co-culture with osteoblast cells. The absorbance values of the four groups increased with the increase of cell culture time. The number of living cells increased as the culture time increased. Insignificant differences in the absorbance values were observed between the 4 groups at 1, 4, and 7 days, thereby showing that the number of living cells at the same time was insignificantly different. When the BaTiO_3_ content of HA/BaTiO_3_ composite piezoelectric ceramics was in the range of 80% to 100%, the composite piezoelectric ceramic had the same biocompatibility as the pure HA. In the experiment, although primary osteoblasts were not induced by osteogenic medium, the ALP enzyme on the sample surface could still be differentiated and synthesized. The ALP activity of HA/BaTiO_3_ piezoelectric bioceramics without loading after co-culture with osteoblasts is shown in Fig. 6(b). The ALP activity of osteoblasts increased as the culture time increased. The ALP activity of osteoblast on HA surface was significantly higher than in the other three groups, and the difference was statistically significant (p < 0.05). When the BaTiO_3_ content of HA/BaTiO_3_ composite piezoelectric ceramics ranged from 80% to 100%, the bone induction activity of the composite was worse than that of HA.

### *In vitro* characterization of HA/BaTiO_3_ piezoelectric bioceramics with cycle loading

**Figure 7:**
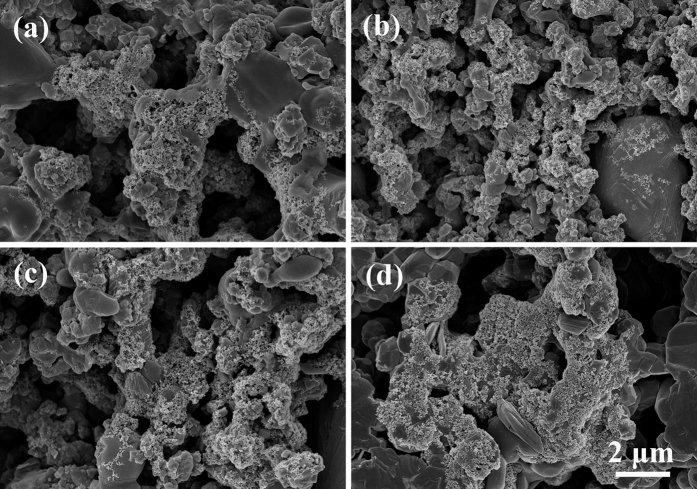
Morphologies of HA/BaTiO_3_ piezoelectric bioceramics after SBF immersion for 7 days with cycle loading. (**a**) 100% BaTiO_3_; (**b**) 90% BaTiO_3_/10% HA; (**c**) 80% BaTiO_3_/20% HA; (**d**) 100% HA.

To simulate the physiological loading of the human body, cyclic loading was applied to the surface of each group. Figure 7 shows the morphologies of HA/BaTiO_3_ piezoelectric bioceramics after SBF immersion for 7 days. After soaking, some particles were deposited on the surfaces of the four groups, which was similar to the result without loading. These particles could be the bone-like apatite. In the dynamic loading process, the electric charge bound on the negative surface of composite piezoelectric ceramics (80% to 100% BaTiO_3_) changed alternately with the piezoelectric properties, and the attraction of the positive ions (Ca^2+^) can also change during circulation. The electric charge attracted the positive ions attached to their negative surface gradually^42^,^43^. The HA in composite piezoelectric ceramics also induced the deposition of bone-like apatite. Thus, the amount of bone-like apatite on the negative surface of composite piezoelectric ceramics was larger than that of the non-loaded bioceramics.

**Figure 8:**
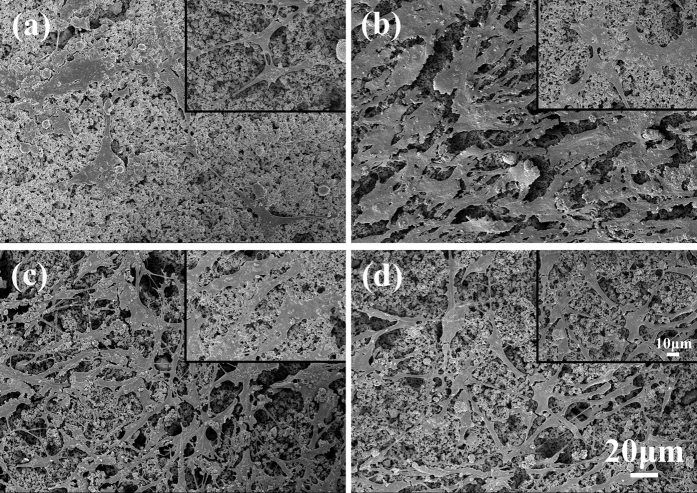
Morphologies of HA/BaTiO_3_ piezoelectric bioceramics with cycle loading after co-culture with osteoblast cells for 3 days. (**a**) 100% BaTiO_3_; (**b**) 90% BaTiO_3_/10% HA; (**c**) 80% BaTiO_3_/20% HA; (**d**) 100% HA. Insert images are localized magnification.

Figure 8 shows the morphologies of HA/BaTiO_3_ piezoelectric bioceramics with cycle loading after co-culture with osteoblast cells for 3 days. The density of osteoblasts on the surface of piezoelectric bioceramics that contained 80% or 90% BaTiO_3_ was significantly higher than that of 100% HA and 100% BaTiO_3_, and the number of pseudopodia was greater. Cells were spread on the surface of the four groups and had pseudopodia (insert images). According to the principle of piezoelectric effect, the piezoelectric material can generate free charge on the surface of the material only under the loading condition. HA/BaTiO_3_ piezoelectric bioceramics are used for bone defects, thereby generating free charge on the surface under loading action. The electric force effect of the bone acted on the microenvironment, which can increase the bone formation protein, β growth factor, and shima II, and affected the deposition of hydroxyapatite on the surface of the samples^44^,^45^,^46^.

**Figure 9:**
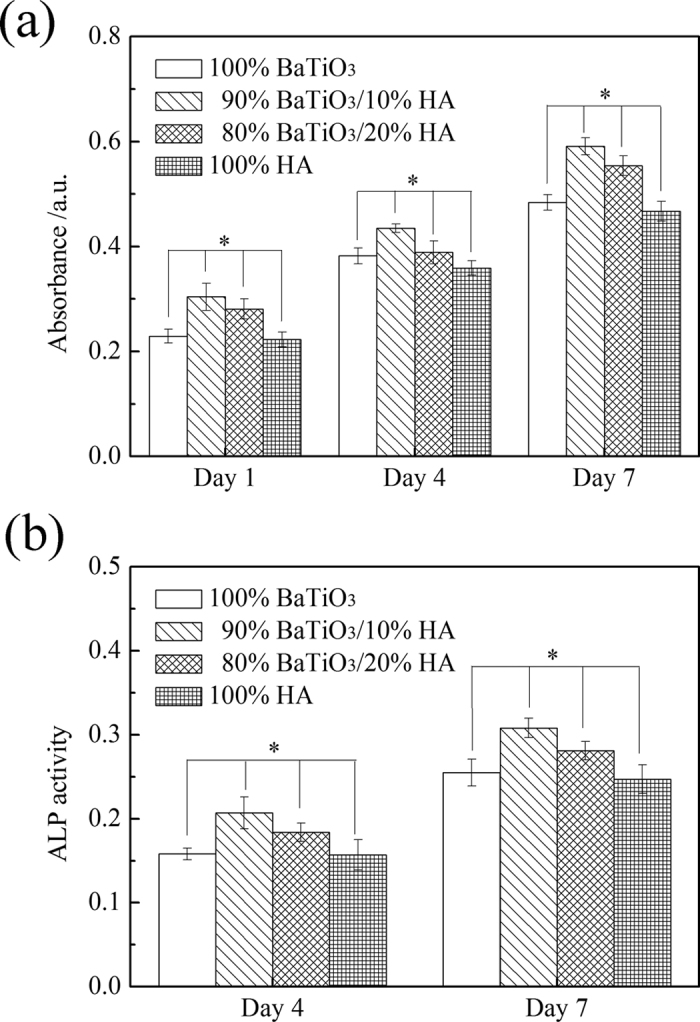
MTT assay and ALP activity of HA/BaTiO_3_ piezoelectric bioceramics with cycle loading after co-culture with osteoblast cells (p < 0.05). (**a**) MTT assay; (**b**) ALP activity.

Figure 9(a) shows the MTT assay of HA/BaTiO_3_ piezoelectric bioceramics with cycle loading after co-culture with osteoblast cells. At different time points, the number of living cells in each group increased as the culture time increased. At the same time point, the number of active cells that contained BaTiO_3_ was more than that of 100% HA. Moreover, the number of living cells was highest for the 90% BaTiO_3_/10% HA piezoelectric bioceramics, and the difference was statistically significant (p < 0.05). This finding showed that the biological compatibility of piezoelectric ceramic that contained BaTiO_3_ was better than that of HA when cycle loading was applied. The electrical stimulation produced by the electric force transformation improved the biocompatibility of BaTiO_3_ when cycle loading was applied on the surface. The biocompatibility of the piezoelectric ceramics increased initially then decreased with the decreasing BaTiO_3_ content. This increase may be attributed to the reduction of piezoelectric properties, which leads to the decrease of biological compatibility caused by electrical stimulation. By contrast, the HA content increased with decreasing BaTiO_3_ content. HA is a material with good biological activity, which will play a positive role in the biocompatibility of the material^47^,^48^. These two trends have different effects on each other, and the 90% BaTiO_3_/10% HA piezoelectric bioceramics have better biocompatibility than the others.

The ALP activity of HA/BaTiO_3_ piezoelectric bioceramics with cycle loading after co-culture with osteoblasts is shown in Fig. 9(b). According to the different time points of the ALP activity, the ALP activity of the cells increased with the increasing culture time. The ALP activity of the piezoelectric ceramics that contained BaTiO_3_ was higher than that of HA. Moreover, the ALP activity of piezoelectric ceramics with 90% content was higher than that of piezoelectric ceramics with 80% and pure BaTiO_3_, and the difference was statistically significant (p < 0.05). This finding showed that the bone induction activity of the piezoelectric ceramic was better than that of HA when cyclic loading was applied. When cyclic loading was applied on HA/BaTiO_3_ piezoelectric bioceramics, the electrical stimulation promoted osteoblast proliferation and growth, which was similar to the actions of piezoelectric effects on human bone growth, molding, and reconstruction. Negative-bound charge with dynamic changes existed on the negative surface of HA/BaTiO_3_ piezoelectric bioceramics, which can absorb positively charged ions in the medium to move toward the negative surface. Positively charged layers were formed gradually, and the protein molecules with negative charges on the surface and cells would be attracted by the positively charged layers. The protein layer can induce the formation of bone-like apatite on the surface of biomaterials, regulate the adsorption of platelets on the material surface, and activate the aggregation function and complement^49^. Furthermore, it can promote the adhesion, proliferation, and differentiation of osteoblasts.

## Conclusions

In summary, BaTiO_3_ with piezoelectric effect was introduced into HA. HA/BaTiO_3_ composites with different compositions were prepared by using the injection molding process, and the piezoelectric properties were obtained by polarization. An *in vitro* biological culture device of piezoelectric material, which can simulate the dynamic environment of the human bone, was designed. The effect of the content of piezoelectric phase on the biological properties was studied by means of apatite deposition and osteoblast culture experiments. Compressive strengths of HA/BaTiO_3_ composites sintered at 1250 °C for 2 h ranged from 16.2 MPa to 28.4 MPa. The *d*_33_ of HA/BaTiO_3_ piezoelectric ceramics after polarization was 1.3 pC/N to 6.8 pC/N. Under static non-loading condition, the change of BaTiO_3_ content had little effect on the biocompatibility and bone-inducing activity of HA/BaTiO_3_ composites, but all composite piezoelectric ceramics were lower than that of pure HA. When cyclic loading was applied on the surface of HA/BaTiO_3_ piezoelectric ceramics, the biocompatibility and bone-inducing activity of HA/BaTiO_3_ piezoelectric ceramics were higher than that of pure HA. The best biocompatibility and bone-inducing activity were demonstrated by the 10%HA/90%BaTiO_3_ piezoelectric ceramics. The deficiency of this experiment is that the results of *in vitro* experiments did not take into account the effects of strength and surface topography on cell adhesion and proliferation in spite of the piezoelectric effect. Further study needs to simulate other movement frequencies and different stresses of the human to examine the role of the piezoelectric effect fully. In addition, the effect of the composite material with a higher piezoelectric coefficient than human bone on cell adhesion and proliferation requires further research.

## Materials and Methods

### Materials

HA (Sigma-Aldrich Chemical Co. Inc., USA) with a size of 0.5 μm to 1.0 μm was used as a biological phase, and BaTiO_3_ powder (<3 μm, Aladdin, Shanghai Aladdin Biochemical Polytron Technologies Inc., China) was used as a piezoelectric phase. Carboxymethyl cellulose (CMC, Tianjin Fuchen Chemicals Co. Ltd., China) was used as a binder. A dispersant (polyacrylate sodium; Sinopharm Chemical Reagent Co., Ltd, China) was used to stabilize the slurry, and deionized water was used as a solvent of slurries.

### Fabrication of HA/BaTiO_3_ composite materials

Composite powders (100 wt.% BaTiO_3_, 90 wt.% BaTiO_3_/10 wt.% HA, 80 wt.% BaTiO_3_/20 wt.% HA, and 100 wt.% HA) were mixed with 1 wt.% of dispersant and 0.5 wt.% binder in deionized water and ground with a ball mill for approximately 48 h. The prepared slurries were poured into plaster cylinders (Φ24 mm × 20 mm). The samples were placed in an unlit place, dried slowly at room temperature, and sintered at 1250 °C in air for 2 h. After cooling, the samples were covered with silver on the upper and lower surfaces, and then the samples were polarized. The polarization field intensity was 0.8 kv/mm to 1.4 kv/mm, the polarization time was 10 min to 50 min, and the polarization temperature was 110 °C to 140 °C. The surface of the sample was polished by using No. 400 sandpaper to remove the silver and to ensure consistent surface roughness of all samples.

### Characterization of materials science

Morphologies of the HA/BaTiO_3_ composite materials were characterized by using a SEM (model JSM-4500, JEOL, Japan). XRD (Model 7000, Shimadzu Limited, Japan) was used to identify the phases in the sintered composite samples. The piezoelectric constant *d*_33_ of the sample was tested by quasi-static *d*_33_ tester (ZJ-3AN, Institute of Acoustics, Chinese Academy of Sciences). The sample size for *d*_33_ testing was Φ10 mm × 2 mm. Samples were measured after polarization and stored at room temperature for 24 h. The positive and negative sides of the samples were indicated. The compressive strength were measured on cylindrical samples of Φ10 mm × 10 mm by using a computer server to control the material testing machine (HT-2402-100KN, Hungta, Taiwan) with a pressure speed of 0.5 mm min^−1^. Five samples were tested to obtain an average value. All samples for experiments are polished to obtain a similar surface roughness.

### Design of *in vitro* dynamic loading device

**Figure 10:**
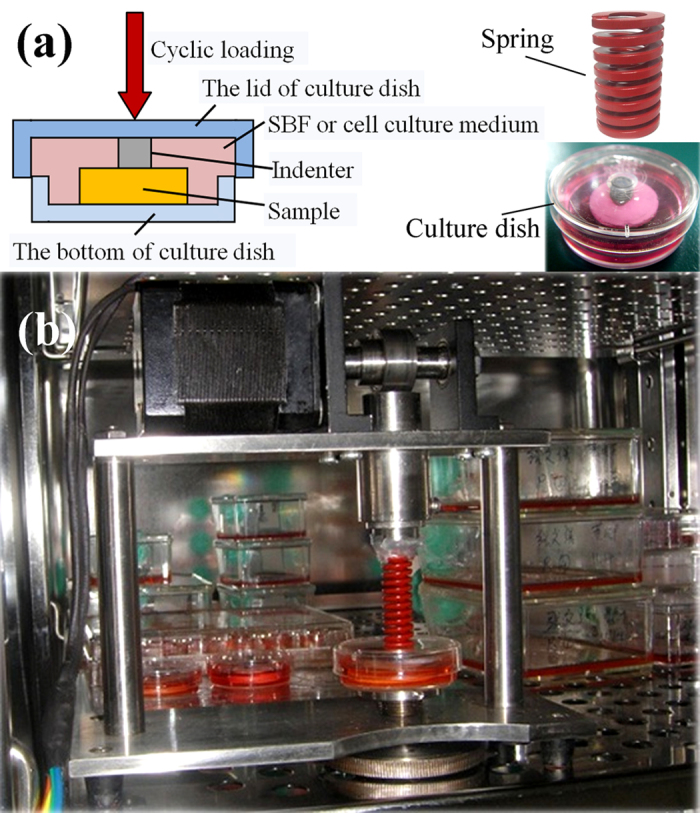
Design of dynamic loading device for piezoelectric bioceramics based on the human activity cycle. (**a**) Design principles; insert images on the right are real photos; (**b**) Dynamic loading device is placed in the incubator. The loading frequency ranges from 0–5 Hz, and the loading force ranges from 0–100 N.

In the experiment, an *in vitro* loading device was designed (Fig. 10). The device consists of a circulating force component, culture dish, and indenter. The working principle of the circulating force component is presented. The outer margin of the rotating cam contacts with the connecting rod, which converts the rotary motion into linear reciprocating motion to form the cyclic displacement. The cyclic displacement was converted into a force with sine distribution by a spring. Force was exerted on the lid of the culture dish, and the inner surface of the culture dish lid was connected with a titanium alloy indenter, which contacts with the composite materials in the culture dish (Fig. 10a). This design has the following advantages: the load cycle of the force can be controlled by the speed of the motor; the value of the force can be adjusted by the elastic coefficient of the spring; the loading curve of the force can be controlled by changing the cam profile; and the indirect transfer of the force ensures that the culture dish will not be contaminated by the loading device. The dynamic loading device was used for *in vitro* biological experiment in the incubator (Fig. 10b) to simulate the physiological loading of bone under different positions of the human body. Loading parameters of 3 Hz and 60 N are selected to simulate the frequency and pressure of the bearing bone when the people walk fast.

### SBF immersion

SBF was prepared according to the ion concentration of Kokubo^50^. The 100 wt.% BaTiO_3_, 90 wt.% BaTiO_3_/10 wt% HA, 80 wt.% BaTiO_3_/20 wt.% HA, and 100 wt.% HA that were static for 24 h after polarization treatment were ultrasonically cleaned in acetone and deionized water. After drying at 70 °C, the samples that were negatively upward were placed in the culture dish, incubated at 37 °C, and soaked for 7 days. The samples were taken out of the SBF and washed with acetone and distilled water. Samples were dried naturally at room temperature. The deposition of HA on the surface of the samples was observed by a SEM (s-4800, Hitachi, Japan). In addition, the samples were loaded using the dynamic loading device for 3 h a day (loading frequency 3 Hz, maximum load 60 N) when soaking in the SBF, and the steps were repeated.

### Cell adhesion

The samples were sterilized with Co-60 then placed in 24-well plates. Osteoblasts were digested into monoplast suspension, and approximately 2 × 10^4^ osteoblasts were seeded on each sample in each well and incubated at 37 °C. The culture medium was changed every 2 days. After culturing for 1, 4, and 7 days, the MTT solution was added to each well and incubated at 37 °C for 4 h. Dimethyl sulfoxide was added to each well, and the solution was transferred to 96-well plates^51^. The absorbance of solutions at 492 nm was measured with an MK-2 microplate reader after culturing for 1, 4, and 7 days. The morphologies of osteoblasts seeded on the samples were observed by SEM. In addition, samples were loaded using the dynamic loading device for 3 h a day (loading frequency 3 Hz, maximum load 60 N) during culture, and the steps were repeated. Experimental results were analyzed using SPSS19.0 software, and p < 0.05 is considered to indicate statistical difference. Five samples of each type were studied. The assays were performed in triplicate.

### Alkaline phosphatase (ALP) activity assay

The 2 × 10^4^ osteoblasts were seeded on each sample in each well and incubated at 37 °C. After culturing for 4 and 7 days, the samples were transferred into new 24-well plates and soaked in 0.3% Triton X-100 to lyse cells after four freezing and thawing cycles. The ALP activity test kit was added to the lysate, and corresponding reagents were added according to the manufacturer’s instructions. The lysate was transferred to 96-well plates, and the absorbance of lysates at 520 nm was measured with the MK-2 microplate reader. In addition, samples were loaded using the dynamic loading device for 3 h a day (The loading frequency is 3 Hz, and the maximum load is 60 N.) during culture, and the steps were repeated. Experimental results were analyzed using SPSS19.0 software, and P < 0.05 is considered to indicate statistical difference. Five samples of each type were studied. The assays were performed in triplicate.

## Additional Information

**How to cite this article**: Tang, Y. *et al*. Fabrication and in vitro biological properties of piezoelectric bioceramics for bone regeneration. *Sci. Rep.* **7**, 43360; doi: 10.1038/srep43360 (2017).

**Publisher's note:** Springer Nature remains neutral with regard to jurisdictional claims in published maps and institutional affiliations.

## Figures and Tables

**Table 1:** Compressive strengthens of HA/BaTiO_3_ composites with different BaTiO_3_ contents.

Composition/wt%	Compressive strength/MPa
100% BaTiO_3_	28.4 ± 3.21
90% BaTiO_3_/10% HA	21.8 ± 2.62
80% BaTiO_3_/20% HA	16.2 ± 1.99
100% HA	21.8 ± 3.01
